# Differential effects of fresh and black garlic extracts on oxidative stress regulation and anti-inflammatory response properties in human HepG2 cells: An *in vitro* study

**DOI:** 10.3389/fphar.2026.1776975

**Published:** 2026-04-07

**Authors:** Manal Almughamisi, Fatimah Amer, Nashi K. Alqahtani, Alaa H. Felemban, Monya T. Jawharji, Halah A. Hafiz, Huda Wazzan, Duaa Altuwairki, Hend F. Alharbi, Reham M. Algheshairy, Rawan Altalhi, Safa H. Qahl, Magbolah S. Alzahrani, Ola A. Abu Ali, Rokayya Sami, Abdullah M. Izmirly

**Affiliations:** 1 Department of Clinical Nutrition, College of Applied Medical Sciences, Taibah University, Medina, Saudi Arabia; 2 Department of Biology, College of Science, King Khalid University, Abha, Saudi Arabia; 3 Department of Food and Nutrition Sciences, College of Agricultural and Food Sciences, King Faisal University, Al-Ahsa, Saudi Arabia; 4 Department of Clinical Nutrition, Faculty of Applied Medical Sciences, Umm Al-Qura University, Makkah, Saudi Arabia; 5 Department of Food and Nutrition, Faculty of Human Sciences and Design, King Abdulaziz University, Jeddah, Saudi Arabia; 6 Department of Food Science and Human Nutrition, College of Agriculture and Food, Qassim University, Buraydah, Saudi Arabia; 7 Department of Biological Sciences, College of Science, University of Jeddah, Jeddah, Saudi Arabia; 8 Department of Biology, Faculty of Science, AL-Baha University, AL-Baha, Saudi Arabia; 9 Department of Chemistry, College of Science, Taif University, Taif, Saudi Arabia; 10 Department of Food Science and Nutrition, College of Sciences, Taif University, Taif, Saudi Arabia; 11 Department of Medical Laboratory Sciences, Faculty of Applied Medical Sciences, King Abdulaziz University, Jeddah, Saudi Arabia; 12 Special Infectious Agents Unit—BSL3, King Fahd Medical Research Center, King Abdulaziz University, Jeddah, Saudi Arabia

**Keywords:** anti-inflammatory, bulbs, garlic, hepatoprotection, lipid, oxidative, peroxidation, stress

## Abstract

Fresh and black garlic exhibits a variety of bioactivities that prevent the development of many diseases. The effect of black garlic bulbs on liver cells was examined in this study. The samples included garlic extracts, ascorbic acid, and atorvastatin in HepG2 cells. Ascorbic acid (94%), atorvastatin (>89%), black garlic (87.29%), and fresh garlic (84.20%) all showed cell viability at 400 μg/mL. Ascorbic acid decreased nitric oxide (NO; 3.23 µM), whereas fresh and black garlic increased it (6.49 and 5.12 µM); all treatments decreased reactive oxygen species (ROS; 0.54–0.76 µM vs. control 1.01 µM) and increased reduced glutathione (GSH; 8.93–10.39 µM vs. control 8.23 µM). Superoxide dismutase (SOD) (21.36 U/mg), catalase (CAT) (28.41 U/mg), and glutathione peroxidase (GPX) (26.14 U/mg) were the antioxidant enzyme activities that were most elevated by black garlic, outperforming ascorbic acid (SOD 20.04, CAT 25.63, and GPX 23.10 U/mg) and fresh garlic (SOD 16.86, CAT 21.47, and GPX 20.10 U/mg). Fresh garlic considerably reduced cholesterol and triglycerides (27.36 and 22.11 μg/mg), black garlic (21.38 and 20.47 μg/mg), and atorvastatin (22.14 and 17.58 μg/mg), but ascorbic acid had no impact (34.02 and 24.54 μg/mg). Black garlic successfully decreased tumor necrosis factor-α (TNF-α) (39.47 pg/mL) compared to the control (48.55 pg/mL), whereas interleukin-1β (IL-1β) values were 30.98 and 41.11 pg/mL, respectively. The results showed that black bulbs may have great effects on oxidative stresses, inflammations, lipid peroxidations, and cell death. According to the present study, black garlic could be a great natural option for developing adjuvant medications and nutritious foods that preserve the liver.

## Introduction

**FIGURE 1:**
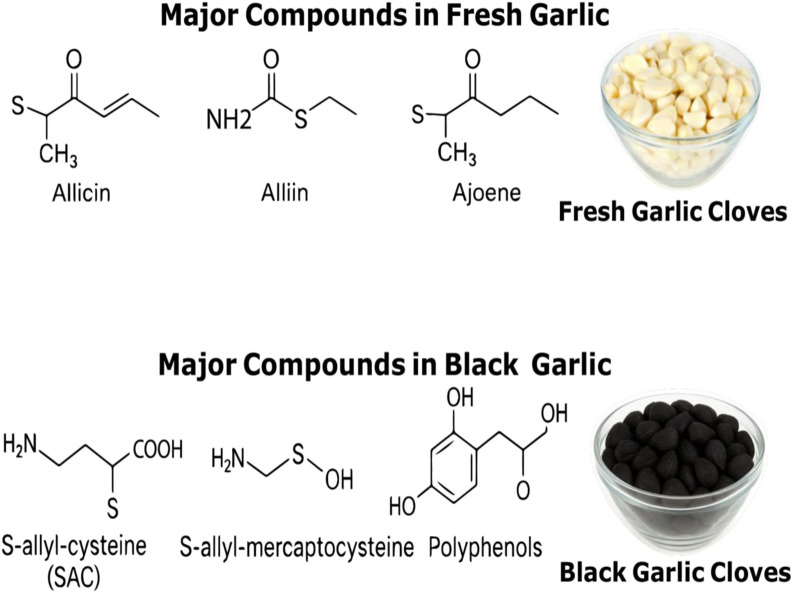
Major chemical components of fresh and aged black garlic.

Garlic bulbs (*Allium sativum*), which belong to the Amaryllidaceae family, have been traditionally used medicinally for several thousand years as an antibiotic. Garlic’s main organosulfur ingredient, allicin, along with saponins and phenols, gives the herb its characteristic flavor and aroma. Allicin’s antioxidant qualities may be the reason for its antibacterial and antiviral efficacies. At doses as low as 1.8 μg, allicin was reported to be able to reduce reactive oxygen species (ROS). The hydroxyl radical by-products decreased by 94% at 36 μg of allicin. Allicin in black garlic breaks down into several organosulfur compounds during processing. Allicin breakdown has little bearing on changes in total phenolic and flavonoid contents, which can be caused by enzymatic activity or by-products of the Maillard reaction, such as 5-hydroxymethyl-2-furfural (HMF), produced after heat treatment (Moses et al., 2024). Although garlic is typically regarded as a health-promoting food, some people may find it difficult to consume due to its strong flavor. Fresh garlic contains γ-glutamyl-S-alk(en)yl-L-cysteine and S-alk(en)yl-L-cysteine sulfoxides, which are the main precursors of S-allylcysteine (Jain et al., 2025). Black garlic is a type of processed food made from fresh garlic that goes through an enzyme-free browning process known as the Maillard reaction when exposed to high heat and humidity for an extended period of time (Nakagawa et al., 2024). As a result of the aging process, which involves fermentation, the chemical composition of the herb changes, and the once-white, bitter garlic cloves turn black and taste sweeter (Mösbauer et al., 2021). Black garlic’s sweet flavor is produced by the breakdown of the organosulfur molecule allicin. Allicin can decompose into a number of antioxidant substances, including flavonoids, sulfur, alkaloids, hydroxymethylfurfural, tetrahydro-β-carbolines, and S-allylcysteine (Yu et al., 2025). Figure 1 presents the chemical components of fresh and aged black garlic.

The quantities of antioxidants increased from 13.91 mg/g to 58.33 mg/g for polyphenol content and from 3.22 mg/g to 15.37 mg/g for flavonoid content as a result of the increase in allicin by-products present in black garlic (Vinayagam et al., 2023). This procedure turns garlic cloves brown to black, giving a delicate texture and an acidic flavor that is similar to that of dried fruits.

Oxidative stress occurs due to an imbalance of ROS in cells or tissues, which disrupts physiological processes. Excessive concentrations of ROS, such as singlet oxygen, hydrogen peroxide, superoxide radicals, and hydroxyl radicals, can harm DNA or cell structure and hinder regular biological processes (Choi et al., 2024).

The liver is the body’s primary digestive organ and is in charge of several detoxifying and metabolic processes, such as nutrition metabolism, lipid and cholesterol homeostasis, and endocrine activity regulation (Abushal et al., 2024). Liver illnesses, such as alcoholic fatty liver, nonalcoholic fatty liver, and chronic hepatitis, can occur as a result of oxidative stress in liver cells caused by exposure to medications, alcohol, or poisons (Alharthi et al., 2025a). Hepatocyte mortality, hepatic stellate cell activation, and lipid peroxidation can all be caused by peroxides generated via mitochondria and peroxisomes (Alharthi et al., 2025b). Phytochemicals, including the many phenolic compounds present in garlic, have a range of physiological benefits, such as anti-inflammatory, antioxidant, and anticancer properties. Black garlic extract may help treat chronic illnesses, lower insulin resistance, and control lipid metabolism (Das et al., 2024; Maeda et al., 2019).

We aimed to study the baseline modulation of fresh and black garlic extracts on the regulation of oxidative stress in human HepG2 cells, compared to ascorbic acid supplementation and atorvastatin treatment.

## Materials and methods

### Garlic samples

White garlic *(Allium sativum*) was purchased from a local grower in Qassim City (2 Kg), Saudi Arabia, and the bulbs were chosen for their consistent size, maturity, and lack of obvious defects. Garlic was harvested in May and preserved at room temperature (∼20 °C) with a relative humidity at 60%–70%.

After being cleaned and peeled, the bulbs were split into two groups. One group was used as fresh white garlic, whereas the other group had controlled fermentation to produce black garlic. The protocol to process black garlic from white garlic was by keeping in a humidity chamber (Model: HCP105, Memmert GmbH, Germany) at temperatures of 70 °C and humidity levels of 80% without any food additives and treatments. Garlic was gradually heated for 35 days before being kept in a thermohygrostatic fermentation chamber to ensure stable humidity and temperature (Fajrani et al., 2021).

The bottom layer of garlic cloves was raised using a bamboo rack to keep them from coming into direct touch with the electric cooker’s base (KH-WA10T; SAMPO, Taoyuan, Taiwan). Fresh garlic cloves were placed on the bamboo rack after the peels were removed. The garlic cloves were layered in the electric cooker and covered with another layer of paper towels. Paper towels were placed over the top layer for avoiding the condensation from pouring onto the cloves throughout the procedure (Yu et al., 2025). Fresh white garlic gradually turned brown due to the Maillard browning reaction, and after a month, it started to slowly darken and soften. Black garlic smells good and tastes smooth and delicious. After that, the lid was closed, and the electric cooker was placed on the warm setting. The black garlic was gathered, cooled to ambient temperature, and stored in sealed containers until additional extraction and examination after it had aged for 35 days.

### Preparation of garlic extraction

Both varieties of garlic were chopped into tiny bits and ground into a powder using a mechanical grinder (MF-10, Jingxin Co., Ltd., China). A volume of 500 mL of distilled water was homogenized with 50 g of garlic samples at a ratio of 1:10 (w/v). The mixture was thoroughly pulverized by blending it. The mixture was placed in a beaker with a magnetic stirrer bar (SB-30PTFE, Shenzhen, China) and swirled for 2 h. Supernatants were extracted after the extracts were centrifuged (GK800-N, Hunan, China) at 5,000 rpm for 20 min at 4 °C to remove the insoluble debris. Water was extracted from the resultant filtrate to guarantee sterility and eliminate particulate matter via a 0.22-µm sterile barrier. The dry matter foundation was used to express all results. A freeze-drying assay was performed after freezing the samples at −80 °C and then using freeze-drying equipment (ALPHA 1-4 LSC, Germany) for 48 h at −50 °C and 0.04 mbar. The dried extracts were kept at approximately −80 °C (Lee et al., 2018).

### Cell culture and study design

The HepG2 model, a human hepatocellular carcinoma cell line from the Shanghai Institute of Biological Sciences (TCHu 72, Shanghai, China), was grown in Dulbecco’s modified Eagle medium (DMEM) with 10% heat-inactivated fetal bovine serum (FBS), 50 U/mL penicillin, 50 μg/mL streptomycin, and 1% L-glutamine. The cell cultures were kept in an incubator with 95% air humidity and 5% CO_2_ at 37 °C (Bontempo et al., 2021; Stormo et al., 2014). Cells were seeded into culture plates and allowed to reach 70%–80% confluence prior to treatment. HepG2 cells were exposed to hydrogen peroxide (H_2_O_2_) at concentrations ranging from 100 to 500 µM for 2–24 h under usual culture conditions to cause hepatocellular damage. The protocol to optimize H_2_O_2_-induced injury comprised extract + H_2_O_2_ and H_2_O_2_-only groups with defined pre-/co-treatment and showed that garlic significantly recovered viability and oxidative/inflammatory indicators compared to H_2_O_2_ alone. An oxidative stress model was created utilizing H_2_O_2_ to assess the hepatoprotective potential of the treatments. The cells were separated into four main groups: H_2_O_2_ (200 µM) and an untreated control. A pretreatment (preventive) approach was used, in which hepatocytes were detected with 200 µM H_2_O_2_ for 4 hours after being incubated for 2 hours with fresh garlic, black garlic, atorvastatin, and ascorbic acid. To provide a constant baseline for evaluating the extracts’ attenuating effects compared to the damage group, this concentration was calibrated to consistently reduce cell viability by approximately 50%.

**FIGURE 2:**
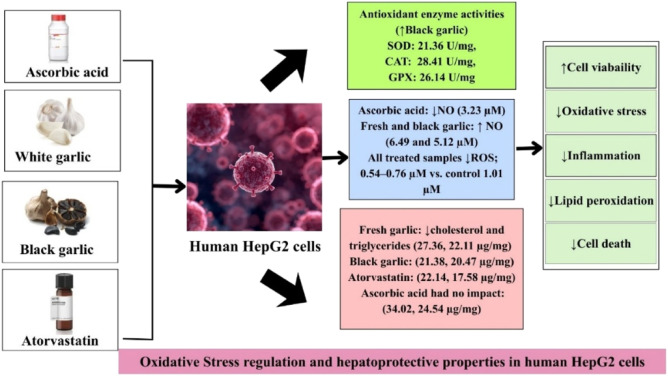
Summary of the experimental design.

Fresh white and black garlic samples (100 μg/mL) (Yu et al., 2025) were compared with ascorbic acid and atorvastatin to evaluate their oxidative stress regulation. Ascorbic acid (A5960), an antioxidant agent, and atorvastatin calcium (A2118), a cholesterol-lowering medication, were obtained/purchased from Sigma-Aldrich. The negative control represents the untreated cells in this study. To ascertain the effects of the treatments on HepG2 cells, a number of assays were carried out after incubation to evaluate cell viability, oxidative stress indicators, lipid metabolism, and inflammatory responses. Figure 2 presents the summary of garlic extracts, ascorbic acid, and atorvastatin by investigating the oxidative stress regulation properties in HepG2 cells.

### Cell viability assay

An MTT assay was used to evaluate cell viability of the garlic samples on HepG2 cells. On 96-well plates, cells were grown at a density of x10^4^ cells/well. After stimulation, each well received 0.5 mg/mL of (MTT) solution (Sigma-Aldrich, United States) in PBS.

The lyophilized garlic extracts and ascorbic acid were dissolved in distilled water to create stock solutions at various concentrations (25, 50, 100, 200, and 400 μg/mL); the cells were cultured overnight, and the mixtures were incubated for 3 hours at 37 °C under 5% CO_2_ for 4 h, which were then further diluted to the appropriate quantities. Atorvastatin was first dissolved in dimethyl sulfoxide (DMSO) and then diluted with culture media to reach the necessary quantities, whereas ascorbic acid was dissolved in distilled water. The treatment medium’s final DMSO content was less than 0.1% (v/v). DMSO solution was added to dissolve the formazan crystals, and a spectrophotometer was used to detect the absorbance at 570 nm with a microplate reader (RT 2100C, Shenzhen, China) (Gao et al., 2019; Sasmaz et al., 2025). Cell viability was represented as a percentage of the control (untreated cells).

### Oxidative stress marker analysis

HepG2 cells were treated with various samples at a dose of 100 μg/mL for 24 h after being seeded at a density of 1 × 10^3^ cells per well. After treatment, cells were processed for oxidative stress measurement and rinsed with phosphate-buffered saline. Several parameters were detected to investigate the oxidative stress markers such as lipid peroxidation, nitric oxide production, ROS, and glutathione content.

### Lipid peroxidation

Lipid peroxidation normally produces malondialdehyde (MDA). On 24-well plates, HepG2 cells were grown in a monolayer. Following three PBS washes, 0.5 mL of cell lysis buffer was added after stimulation. Following the manufacturer’s instructions, an MDA assay kit (MAK085, Sigma-Aldrich) was used to determine the MDA contents. The method is based on the formation of a red thiobarbituric acid reactive substance (TBARS) complex, which is expressed as MDA content. Cell lysate (200 µL) was reacted with thiobarbituric acid, and MDA reacted under acidic condition at high temperature (95 °C) for 45 min, followed by centrifugation at 2,500 rpm for 10 min (Shalaby et al., 2021). The absorbance of TBARS was evaluated colorimetrically at 532 nm, whereas the results were expressed as nmol/mg.

### Nitric oxide (NO) production

The Griess reaction was used to evaluate nitrite. A volume of 100 μL of medium was combined with an equivalent volume of Griess reagent (Sigma-Aldrich, Germany) (0.5% sulfanilamide, 2.5% H_3_PO_4_, and 0.05% naphthyl ethylene diamine in H_2_O), and the mixture was allowed to sit at ambient temperature for 15 min in the darkness. Absorbance was measured at 550 nm against sodium nitrite, whereas the results were expressed in µM (Bontempo et al., 2021).

### Reactive oxygen species

ROS levels were determined using a microplate reader to quantify fluorescence after the cells were incubated with 10 µM of the 2′,7′-dichlorofluorescein diacetate (DCFH-DA) kit (D6883, Sigma-Aldrich) for 30 min at 37 °C in the dark. PBS was used to wash the cells once more after any excess dye had been removed. Fluorescence intensity at a 485/538 nm filter was measured using a microplate reader (Ali et al., 2017). ROS data were expressed as normalized fluorescence (% of control). The results were expressed as mean ± standard deviation (SD).

### Glutathione content (GSH)

A colorimetric test based on the interaction of free sulfhydryl groups with Ellman’s reagent [5,5′-dithiobis-(2-nitrobenzoic acid) (DTNB)] was used to measure the reduced glutathione (GSH) concentration using the (CS0260) kit from Sigma-Aldrich. After preparing cell lysates, an aliquot of 100 µL was combined with 50 µL of DTNB reagent for each sample and allowed to sit at room temperature for 10 min. Using a microplate reader, the production of the yellow-colored 5-thio-2-nitrobenzoic acid (TNB) was quantified spectrophotometrically at 412 nm against glutathione (Tsai et al., 2019). Glutathione concentrations were expressed in µM.

### Antioxidant defense enzymes

Superoxide dismutase (SOD) which converts superoxide radicals into oxygen and H_2_O_2_, catalase (CAT) which breaks down H_2_O_2_, and glutathione peroxidase (GPX) which converts NADPH substrates to NADP were chemically detected in triplicate using the commercial assay kits (MAK196, MAK080, and CGP1 from Sigma-Aldrich), in line with the manufacturer’s guidelines by using a microplate reader at 450 nm, 520 nm, and 340 nm, respectively. Results from the spectrophotometric method were expressed in U/mg (Lee et al., 2009).

### Lipid metabolism markers

HepG2 cells’ intracellular triglyceride kit which hydrolyzes triglycerides to glycerol and cholesterol kit based on enzymatic conversion to a colored end product were measured using colorimetric enzymatic assays (MAK043 and MAK266 kits from Sigma-Aldrich) in accordance with the manufacturer’s instructions. HepG2 cells were seeded in 6-well culture plates with 2 mL of complete DMEM at a density of 2 × 10^3^ cells/well, and they were left to adhere overnight. The prescribed chemicals were then added to the cells at the appropriate concentrations for the duration of the incubation period. Following treatment, cells were lysed using either a conventional nonionic lysis buffer as Tween-20 based buffer with 300 µL of the lysis solution after being twice washed with ice-cold PBS to remove extracellular lipids. After15 minutes of ice incubation, cell lysates were centrifuged at 15,000 rpm for 10 min at 4 °C to eliminate cellular debris. Lipid analysis was performed on the resultant supernatants. Triglyceride content and total cholesterol levels were detected at 570 nm and 550 nm using a microplate reader, whereas results were expressed in µg/mg (Kim et al., 2022).

### Cytokine markers

HepG2 cells were suspended in DMEM supplemented with 10% FBS after being adjusted to a density of 5.0 × 10^2^ cells/well. For 48 h, the cells were incubated at 37 °C in a humidified environment with 5% CO_2_. After incubation, culture supernatants were collected for cytokine quantification using commercial ELISA kits, such as tumor necrosis factor-alpha (TNF-α), interleukin-6 (IL-6), and interleukin-1 beta (IL-1β) (RAB0308, RAB0477, and RAB0304, Sigma-Aldrich, United States). Using an ELISA microplate reader (BK EL10A, Shandong, China), absorbance was evaluated at 450 nm, whereas results were expressed as pg/mL (Zhong et al., 2022).

### Statistical analysis

There were three independent studies, all of which used biological duplicates and were carried out in HepG2 cells that had been cultivated separately. Measurements were taken from technical triplicates for every biological replicate, and statistical analysis was performed using the mean value. Information is displayed as mean ± SD. The values from each group were compared using one-way analysis of variance (ANOVA) with Tukey’s honestly significant difference (HSD) *post hoc* test. SAS (version 8.02) was used to perform a statistical analysis. The data were analyzed using GraphPad Prism. P-values less than 0.05 were regarded as statistically significant.

## Results and discussion

### Cell viability in HepG2 cells

**FIGURE 3:**
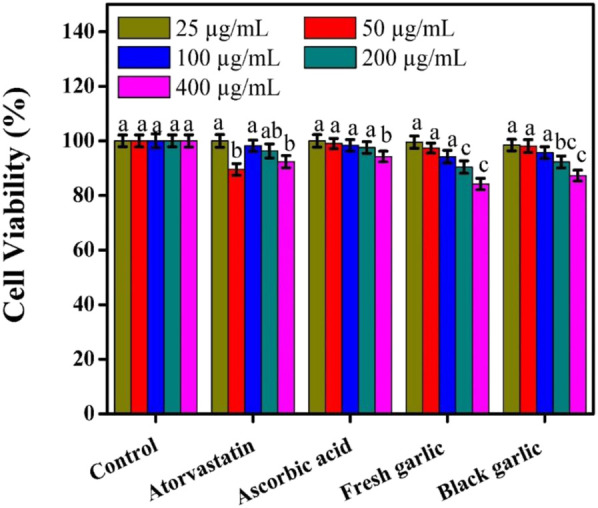
Cell viability counts among groups in HepG2 cells. The mean ± SD of the three independent biological tests (n = 3) with technical triplicate measurements is represented by the data. Garlic extracts showed unique biological activities when compared to ascorbic acid and atorvastatin, which may contribute to their antioxidant potentials.

HepG2 cell viability was assessed at dosages ranging from 25 to 400 μg/mL of fresh garlic, black garlic, ascorbic acid, and atorvastatin, Figure 3. Normal cell growth and assay reliability were confirmed by the untreated control cells’ 100% vitality at all tested doses. Ascorbic acid’s well-known antioxidant and cytoprotective qualities were demonstrated by its minimal cytotoxicity, with cell viability persisting over 94% even at the maximum dose (400 μg/mL). Atorvastatin’s relatively mild cytotoxic effects validated its properties at moderate doses, with survival values over 89% at all dosages. Compared to black garlic, fresh garlic extract reduced cell viability, especially at higher concentrations. Black garlic preserved a somewhat greater viability of 87.29%, but fresh garlic decreased cell viability to 84.20% at 400 μg/mL. These findings appear to be agreed with those previously published by Sasmaz et al. (2025), who focused on the comparative phytochemicals and bioactive components of fresh and aged garlic samples. This discrepancy showed that fermentation would have reduced some cytotoxic components while maintaining black garlic’s antioxidant activity (Bontempo et al., 2021). Fresh garlic may have a greater impact because it includes more reactive organosulfur compounds, such as allicin, which affect cellular metabolism and oxidative stress pathways (Lee et al., 2018). Black garlic seems to have a more balanced effect, lowering oxidative stress without significantly impairing cell viability because it is rich in long-lasting antioxidant chemicals that are produced during fermentation and have antiviral effects on herpes simplex virus-2 in lung cells (Horowitz et al., 2024). Atorvastatin, a popular hepatoprotective drug, and garlic extracts also showed a variety of benefits. Garlic extracts demonstrated a slight decrease in cell viability, indicating cellular stress modulation rather than overt toxicity, whereas atorvastatin maintained high cell viability, consistent with its protective activity. The results confirmed that both fresh and aged garlic extracts had concentration-dependent impacts on HepG2 cell viability, with fresh garlic having considerably greater effects. Similar results of cell viability on HepG2 cells were observed by Ngan et al. (2017), who studied the biological activity of aged garlic fermented with *lactobacillus plantarum* PN05 and various aged garlic types.

### Oxidative stress markers in HepG2 cells

MDA is the final by-product of lipid peroxidation and was created when free radicals attacked the plasma membrane. Furthermore, MDA has been employed extensively as a lipid peroxidation damage indicator (Tsai et al., 2019). Numerous critical processes, such as gene expression, differentiation, the cell cycle, and death, are regulated by NO, which is generated by the NO synthase (NOS) enzymes (Bontempo et al., 2021).

Proteins, lipids, and DNA are capable of holding oxidative damage from ROS, which are naturally occurring by-products of cellular metabolism (Ali et al., 2017). GSH is an important nonenzymatic antioxidant that can be oxidized to glutathione disulfide (GSSG), which reduces the toxicity of hydrogen peroxide, hydroperoxide, and xenobiotics. Both GSH-Px and GSH-Rd are GSH-related enzymes that aid in detoxification, even though antioxidant action in cellular defense entails conjugation with glutathione or the removal of free radicals (Tsai et al., 2019).

**FIGURE 4:**
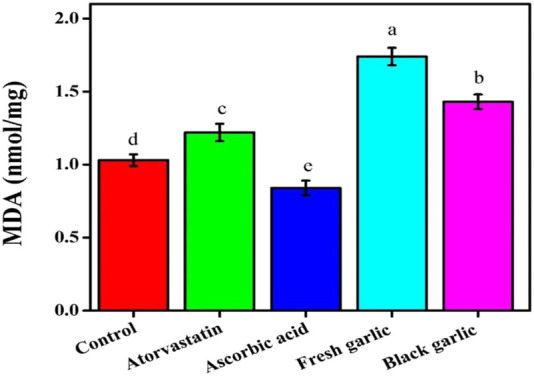
Lipid peroxidation levels among groups in HepG2 cells. The mean ± SD of the three independent biological tests (n = 3) with technical triplicate measurements is represented by the data.

Fresh garlic treatment dramatically increased MDA levels (1.74 nmol/mg) compared to control cells (1.03 nmol/mg); black garlic treatment also increased MDA levels (1.43 nmol/mg), but in reduced degree, Figure 4. Similar results on MDA levels were observed by Ahmed (2018), who studied aged black garlic’s antiapoptotic properties against cyclophosphamide-induced hepatotoxicity. The increased MDA indicates a modulation of oxidative stress under experimental settings and should be read with supporting data, not as a direct pro-oxidant effect of the extracts.

**FIGURE 5:**
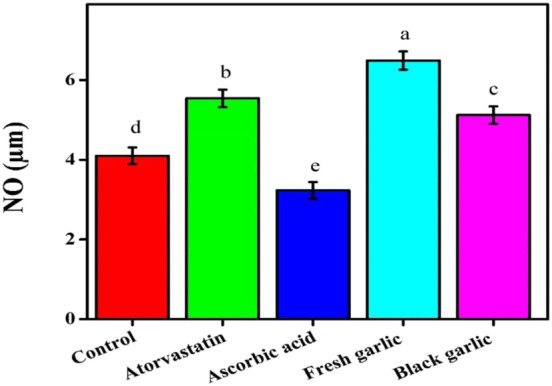
Nitric oxide production levels among groups in HepG2 cells. The mean ± SD of the three independent biological tests (n = 3) with technical triplicate measurements is represented by the data.

**FIGURE 6:**
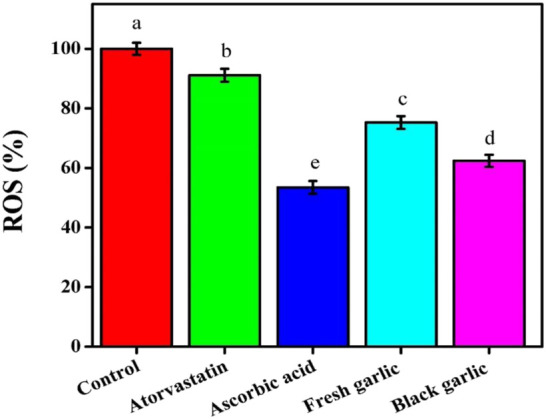
Reactive oxygen species among groups in HepG2 cells. The mean ± SD of the three independent biological tests (n = 3) with technical triplicate measurements is represented by the data.

Ascorbic acid administration decreased MDA levels to 0.84 nmol/mg, demonstrating its strong antioxidative impact (Lee et al., 2018). Both garlic extracts increased NO generation, with fresh garlic exhibiting the largest increase (6.49 µM), followed by black garlic (5.12 µM), whereas ascorbic acid reduced NO levels to 3.23 µM, Figure 5. Compared to the control, ROS levels were reduced by all treatments; ascorbic acid showed the largest reduction (53.47%), followed by fresh garlic (75.25%) and black garlic (62.38%), Figure 6.

**FIGURE 7:**
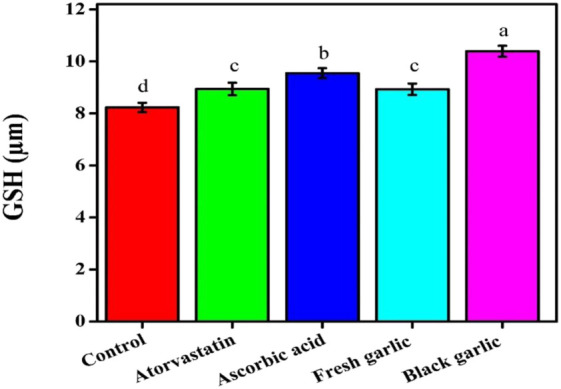
Glutathione content among groups in HepG2 cells. The mean ± SD of the three independent biological tests (n = 3) with technical triplicate measurements is represented by the data.

The cells treated with black garlic (10.39 µM) and ascorbic acid (9.55 µM) had higher levels of GSH, an important intracellular antioxidant, than the control group (8.23 µM), Figure 7. Both atorvastatin (8.94 µM) and fresh garlic (8.93 µM) marginally increased GSH levels.

Both fresh and black garlic extracts changed oxidative stress indicators in HepG2 cells. Fresh garlic increased MDA and NO levels, indicating its function in enhancing cellular defense mechanisms and regulating redox signaling (Shalaby et al., 2021). Similar results on NO levels were obtained by Ishikawa et al. (2006), who focused on how aged garlic extracts affect cancer cells. A more balanced response that greatly increased GSH content, with a slight increase in MDA and NO levels, demonstrated black garlic’s antioxidative potential (Fajrani et al., 2021). Similar findings on GSH levels were reported by Shalaby et al. (2021), who examined the mechanisms of both garlic samples’ hepatoprotective properties against N-nitrosodimethylamine-induced preneoplastic lesions in rats. The treatments dramatically changed ROS levels, with aged garlic extract lowering intracellular ROS formation compared to control cells. It is interesting to note that black garlic outperformed atorvastatin in increasing GSH levels, indicating that it strengthens intracellular antioxidant defenses more effectively than the medication. Several biological processes can be reflected in higher NO levels. NO may contribute to nitrosative stress, especially through inducible nitric oxide synthase (iNOS) activity and the subsequent generation of peroxynitrite, even if it can also function as a signaling molecule and have protective benefits in some situations (Bontempo et al., 2021). The data showed that garlic extracts had the ability to modulate organic oxidative stress in human liver cells.

### Antioxidant defense enzymes in HepG2 cells

**FIGURE 8:**
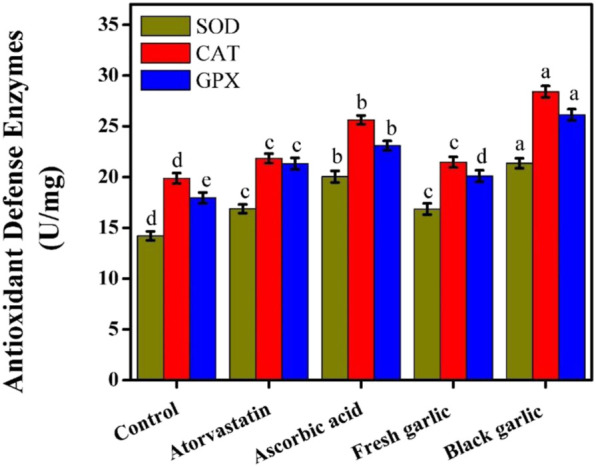
Antioxidant defense enzymes among groups in HepG2 cells. The mean ± SD of the three independent biological tests (n = 3) with technical triplicate measurements is represented by the data. This demonstrated the potential of black garlic as a dietary supplement or supplemental treatment agent for liver illnesses caused by oxidative stress.

The current results showed that black garlic was more effective than fresh garlic at modulating antioxidant defense enzymes in HepG2 cells. The bioactive components of black garlic may work together to improve cellular antioxidant capacity and control oxidative stress, as indicated by the notable overexpression of SOD, CAT, and GPX after treatment (Lee et al., 2018). Given that aging is known to increase the levels of stable, organosulfur compounds such as S-allyl cysteine, aged garlic may have better antioxidant response than fresh garlic (Tsai et al., 2019). By boosting the synthesis and activity of antioxidant enzymes, these substances increase resistance against oxidative damage. After HepG2 cells were treated with fresh garlic, black garlic, ascorbic acid, and atorvastatin, antioxidant defense enzyme activities were assessed. All treatments increased SOD, CAT, and GPX activities to varying degrees compared to control cells. Antioxidant enzyme activities were most increased by black garlic treatment, with SOD, CAT, and GPX levels reaching 21.36, 28.41, and 26.14 U/mg, respectively. These results showed a greater activation of endogenous antioxidant defenses than those detected in the ascorbic acid-treated group. Compared to the control, ascorbic acid markedly increased the activities of SOD (20.04 U/mg), CAT (25.63 U/mg), and GPX (23.10 U/mg), Figure 8. Fresh garlic increased antioxidant enzyme activities compared to untreated cells. When compared to the control cells, atorvastatin somewhat increased antioxidant enzyme activities; nevertheless, its effects were still less than those of ascorbic acid and black garlic. Similar results were observed by Shalaby et al. (2021), who studied white and black garlic extracts in an animal study. Extracts from white and black garlic improved the hepatic antioxidant defense system by increasing important antioxidant enzymes, which decreased oxidative stress and shielded liver tissue from damage caused by N-nitrosodimethylamine.

Ageing entire garlic can increase antioxidant activity, as evidenced by the antioxidant value of fermented aged garlic being (4.5 times) higher than that of fresh garlic (Lee et al., 2009). This outcome was consistent with other study that revealed that aged black garlic exhibited 3–9 times higher SOD activity *in vitro* at concentrations of 20–100 mg/mL (Jung et al., 2006). γ-Glutamyl cysteine, which may be hydrolyzed and oxidized to form alliin, is the main sulfur-containing compound found in undamaged garlic bulbs (Amagase, 2006). After alliin is crushed, chopped, chewed, or dehydrated, alliinase transforms it into odoriferous thiosulfinate allicin. Similar findings were found by Ahmed (2018), who examined the hepatic enzymes of black and white garlic extracts in rat models compared to cyclophosphamide, which increased the antioxidant defense enzymes (SOD, CAT, and GSH).

Black garlic exhibited equivalent or greater effects on antioxidant enzyme activation compared with ascorbic acid, suggesting that it functions as both a direct antioxidant and a modulator of endogenous defense systems (Amagase et al., 2001). Atorvastatin’s major pharmacological role as a lipid-lowering medicine may have hampered its ability to fully battle oxidative stress, despite its mild antioxidant capabilities. However, the natural bioactive components of garlic have demonstrated wider cytoprotective advantages, especially when fermented. These findings showed that black garlic outperformed fresh garlic, ascorbic acid, and atorvastatin in HepG2 cells, exhibiting distinct and synergistic antioxidant properties.

### Lipid metabolism markers in HepG2 cells

**FIGURE 9:**
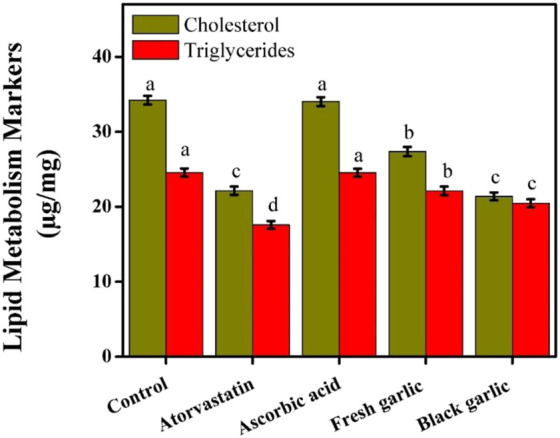
Lipid metabolism markers among groups in HepG2 cells. The mean ± SD of the three independent biological tests (n = 3) with technical triplicate measurements is represented by the data. Black garlic might be used as a functional food or additional medication to address oxidative stress-related hepatic lipid issues.

The current findings showed that both fresh and black garlic extracts affected the lipid metabolism of HepG2 cells, with black garlic having a stronger lipid-lowering effect. The decrease in intracellular triglycerides and cholesterol detected after treatment increases the possibility that black garlic’s bioactive ingredients work together to regulate hepatic lipid homeostasis. The levels of cholesterol and triglycerides in control cells were 34.22 μg/mg and 24.55 μg/mg, respectively. Atorvastatin treatment dramatically decreased intracellular lipid accumulation, with cholesterol and triglyceride levels decreasing to 22.14 and 17.58 μg/mg, respectively. Black garlic treatment reduced lipid levels similarly to atorvastatin, with cholesterol and triglyceride values of 21.38 and 20.47 μg/mg, respectively. Fresh garlic decreased intracellular triglycerides (22.11 μg/mg) and cholesterol (27.36 μg/mg) compared to control cells, Figure 9. Ascorbic acid had no discernible effect on lipid metabolism; triglyceride and cholesterol levels (34.02 and 24.54 μg/mg, respectively) were similar to those of the control group. Similar results for the lipid profile were observed by Nurmawati et al. (2021), who studied the effects of single-clove black garlic on the lipid profile and hemostasis status in male rats with nonalcoholic fatty liver disease. Black garlic has hepatoprotective potential, as its lipid-lowering effectiveness was comparable to that of atorvastatin, a clinically used lipid-lowering drug. The current results were in agreement with the previous findings by Shin et al. (2014), who studied the aged black garlic extract’s hepatoprotective effect on rodents. Black garlic may have a combination of antioxidant action and modification of lipid metabolic pathways, offering wider cellular protection, whereas atorvastatin successfully decreased lipid buildup by inhibiting cholesterol production. In a follow-up study, Villaño et al. (2024) examined the effects of aged garlic consumption on lipid profiles and endothelial functioning with and without excessive cholesterol.

The aging process, which increases the content of stable organosulfur compounds and polyphenols known to affect lipid metabolism and oxidative stress regulation, may have contributed to this discrepancy (Bontempo et al., 2021). Garlic’s flavonoid can reduce lipid profile levels by acting as an antioxidant and preventing the synthesis of triglycerides and total cholesterol (Prihanti et al., 2019).

However, ascorbic acid supplementation had no discernible effect on triglyceride or cholesterol levels, suggesting that its function was mostly restricted to antioxidant activity rather than direct control of lipid metabolism. This differentiation emphasized the special benefit of garlic extracts, especially black garlic, in lowering fat increase and oxidative stress. These results showed that black garlic had distinct and combined effects on hepatoprotection and lipid metabolism in HepG2 cells, outperforming fresh garlic and ascorbic acid and nearly equaling the effectiveness of atorvastatin in decreasing cholesterol.

### Cytokine markers in HepG2 cells

**FIGURE 10:**
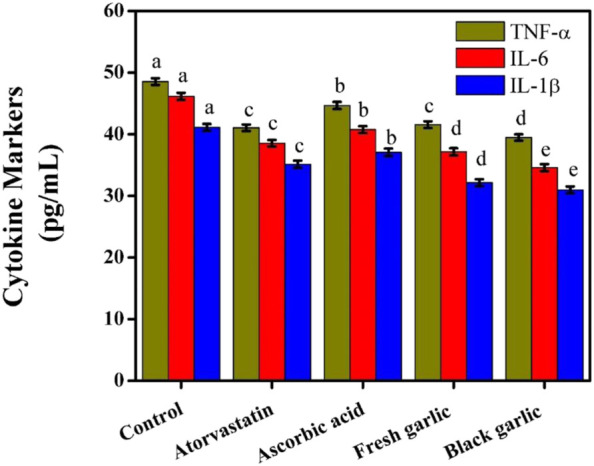
Cytokine markers among groups in HepG2 cells. The mean ± SD of the three independent biological tests (n = 3) with technical triplicate measurements is represented by the data. These results validated the use of black garlic as an additional treatment approach or as part of a functional diet for liver illnesses associated with oxidative stress and inflammation.

The current results showed that HepG2 cells’ production of inflammatory cytokines was successfully regulated by both fresh and black garlic extracts, with black garlic having more anti-inflammatory activity. The decrease in TNF-α, IL-6, and IL-1β after black garlic administration indicated that its bioactive ingredients worked in concert to reduce inflammatory signals related to oxidative stress. The baseline levels of TNF-α (48.55 pg/mL), IL-6 (46.15 pg/mL), and IL-1β (41.11 pg/mL) were higher in control cells, Figure 10. When compared to the control, all treatments decreased the generation of cytokines. The aged garlic resulted in the most pronounced reduction in inflammatory markers, with TNF-α, IL-6, and IL-1β levels decreasing to 39.47, 34.58, and 30.98 pg/mL, respectively.

Although the reductions were less pronounced than those shown with black garlic, fresh garlic still considerably lowered cytokine levels, especially IL-6 (37.14 pg/mL) and IL-1β (32.14 pg/mL). Pro-inflammatory cytokines were reduced by atorvastatin treatment, with TNF-α, IL-6, and IL-1β values of 41.05, 38.54, and 35.14 pg/mL, respectively. Compared to the control, ascorbic acid supplementation reduced cytokine levels; however, its effects were less pronounced than those of both garlic extracts, especially for IL-1β. The aging process, which increases the quantity of persistent antioxidant and anti-inflammatory chemicals, including S-allyl cysteine and polyphenols, may be responsible for the greater cytokine suppression detected with black garlic as opposed to fresh garlic. By inhibiting redox-sensitive inflammatory pathways, these substances lower cytokine expression (Shalaby et al., 2021). Compared to ascorbic acid, black garlic demonstrated a greater ability to regulate inflammatory mediators, indicating that its protective properties extended beyond scavenging free radicals to include regulating inflammatory reactions. This showed how ineffective ascorbic acid supplementation is at lowering hepatic inflammation achieved by cytokines. After consumption of fresh and black garlic, the production of TNF and IL-1 dramatically decreased (Tsai et al., 2019). Despite atorvastatin’s well-established medical application, black garlic showed greater anti-inflammatory qualities. Through indirect anti-inflammatory pathways, atorvastatin decreased cytokine levels; however, its effects were not as strong as those of black garlic (Lee et al., 2018; Prihanti et al., 2019). This demonstrated that by addressing oxidative stress and inflammatory pathways at the same time, garlic extracts—especially black garlic—provided wider protection. These findings showed that black garlic outperformed fresh garlic, ascorbic acid, and atorvastatin in exerting distinct and synergistic anti-inflammatory effects in HepG2 cells.

## Conclusion

The study’s findings suggest that fresh and black garlic may have preventive properties. Black garlic contains a variety of bioactivities that aid in preventing the onset of many diseases. These findings could advance our knowledge of how garlic protects the liver by boosting antioxidant enzyme activity and halting lipid peroxidation. Further *in vivo* research is needed to confirm the possible health advantages of black garlic to protect liver cells.

### Strengths, limitations, and future perspectives

This work added important experimental evidence to the expanding literature of research on garlic-derived functional foods and nutraceuticals by comparing the regulation of oxidative stress by fresh and black garlic extracts. The continuous evaluation of several oxidative stress biomarkers was a major strength of the current study, enabling a more thorough comprehension of the redox-modulating properties of garlic extracts. The identification of long-term safety profiles and ideal consumption dosages for both fresh and black garlic remained a crucial issue for future research. These results could enhance future drug design techniques based on bioactives obtained from garlic and aid in the creation of targeted therapeutic formulations. The content of phenolics, flavonoids, and other bioactives in 100 μg/mL fresh and 100 μg/mL black garlic extracts cannot be confirmed because extraction yield, total phenolic content and total flavonoid were not quantified. Black garlic has more pronounced and unique phenolic and flavonoid profiles than fresh garlic. Dose comparison is therefore purely mass-based and should not be considered chemically similar, but rather exploratory. The comparisons were based on crude extract mass and were exploratory.

## Data Availability

The original contributions presented in the study are included in the article/Supplementary Material, further inquiries can be directed to the corresponding author.
